# Contamination of health care workers’ coats at the University Teaching Hospital in Lusaka, Zambia: the nosocomial risk

**DOI:** 10.1186/s12995-015-0077-2

**Published:** 2015-09-16

**Authors:** Susan Mwamungule, Henry Mwelwa Chimana, Sydney Malama, Geoffrey Mainda, Geoffrey Kwenda, John Bwalya Muma

**Affiliations:** Department of Biomedical Sciences, School of Medicine, University of Zambia, Lusaka, Zambia; Health Promotions Unit, Institute of Economic and Social Research, University of Zambia, Lusaka, Zambia; Department of Disease Control, School of Veterinary Medicine, University of Zambia, Lusaka, Zambia

**Keywords:** Health-worker, Nosocomial, Bacteria, Antibiotic- resistance, Zambia

## Abstract

**Background:**

Health care Associated Infections (HAIs) are a major public health problem in both developed and developing countries. They pose a severe impact in resource-poor settings, where the rate of infection is estimated to be relatively high. Therefore, this study was conducted to establish empirical evidence related to HAIs in Zambia.

**Method:**

This was a prospective cross-sectional study conducted from October, 2013 to May 2014 at the University Teaching Hospital (UTH) in Lusaka. A total of 107 white coats worn by health care-workers at UTH were sampled for possible bacteriological contamination.

**Results:**

Of the 107 white coats screened, 94 (72.8 %) were contaminated with bacteria. There was no difference in the contamination levels between white coats worn for more than 60 min (47.8 %) compared to those worn for 30–60 min (46.7 %) (*p* = 0.612). Further, the antibiotic sensitivity tests indicated that the bacterial isolates were resistant to some of the antibiotics assessed. Isolates of *Staphylococcus aureus* and *Klebsiella pnumoniae* exhibited the highest resistance to most of the antibiotics assessed.

**Conclusion:**

This study has shown that white coats worn by health care-workers at the University Teaching Hospital generally have high microbial contaminations and hence pose a nosocomial risk. It is therefore, recommended that white coats be regularly sanitized, and health care workers also be sensitized on public health risk of HAIs associated with contaminated coats.

## Introduction

Health-care Associated Infections (HAIs) are a major public health problem in both developed and developing countries [1, 2]. They pose a severe impact in resource-poor settings, where the rate of infection is estimated to range from 25 to 40 % (WHO 2005, 2008). Studies have shown that HAIs exert a tremendous toll on patients, families and systems of care, resulting in increased morbidity and mortality, and eventually increasing healthcare costs [2]. An estimated 1.4 million people worldwide suffer from infections acquired in hospitals [3]. Prevention of health care associated infections has increasingly received attention in recent years due to the ever–increasing health care costs, as well as the escalating problem of antibiotic resistance [4]. Previous studies have shown that up to 37 % of nosocomial infections in intensive care units (ICUs) are directly attributable to transmission of resistant organisms across patients [5]. Standard infection control practices have focused on universal hand hygiene and barrier precautions for patients infected or colonized with resistant organisms.

The role of bacterial contamination of uniforms of health care workers (HCWs) in the horizontal transmission of bacteria remains poorly understood. Despite their efforts, healthcare workers may serve as potential vectors of disease, disseminating virulent microorganisms among their patients [6]. Health care workers are at risk of contracting infectious microorganisms by virtue of being in constant contact with patients. Therefore, both patients and health care workers can transmit infection through direct contact, as well as through indirect contact with inanimate objects.

In 2005, WHO Patient Safety Initiative launched the First Global Patient Safety Challenge to galvanize international focus and action on the critical issue of HAIs. Since then, there has been a growing body of data implicating HCWs’ uniforms as a potential reservoir of pathogenic organisms [7].

A recent study in Israel isolated pathogenic bacteria on uniforms of 85 of 135 (63 %) physicians and nurses [8]. These contaminated uniforms might have acted as vectors for the continued dissemination of bacteria from patients to HCWs and vice versa. Healthcare-associated infections represent a heavy social and financial burden. Another study on white coats reported 95 % contamination, while others found presence of *Staphylococcus aureus* in 23 % of the white coats [8, 9]. These bacteria appear to be found more specifically around sleeves and pockets [10]. In the USA, it was estimated that 1.7 million cases of HAIs occurred every year, with almost 99,000 deaths related to these cases [10].

The WHO Patient-Safety Initiative indicated that any potential source of HAIs that could threaten the well-being of individuals within healthcare facilities should be investigated and mitigated (WHO, 2005). There is however, no documented evidence indicating that similar studies have been done in Zambia. Therefore, this study was conducted to establish empirical evidence related to HAIs in Zambia.

## Materials and methods

### Study site and design

This was a prospective cross-sectional study conducted at the University Teaching Hospital (UTH) in Lusaka from October, 2013 to May 2014. The hospital is the largest hospital in Zambia and often serves as a national referral center for people needing specialized medical treatment.

### Study population

The study population included health care workers at UTH from the following departments based on their unique mode and levels of contamination: Obstetrics and Gynaecology, the Outpatient (OPD), Pathology & Microbiology, Intensive Care Unit, Medical, Paediatrics and Surgical wards.

### Inclusion and exclusion criteria

The study included health workers coming in direct contact with patients who wore white coats from the laboratory, intensive care unit, and paediatrics, surgical, obstetrics and gynecology and OPD departments. Health workers who wore white coats but did not deal directly with patients were excluded from the study. It was estimated there were approximately 133 persons in the selected units who wore white coats and came in direct contact with patients which thus form our study population.

### Sample size

There was no data on the prevalence of microbiological contamination of health workers coats in Zambia. Therefore, in order to estimate the prevalence within 5 % allowable error and considering a 95 % confidence level, the calculation was based on the assumption that 50 % of health workers’ coats in Zambia had microbiological contamination. Based on these assumptions, the number of persons to be sampled were estimated using the simple random formula as indicated by Dohoo et al. [11]. Therefore, the number of persons to be included in the study was calculated to be 384 health workers, given that sampling would be done from an infinite population. This sample size was adjusted taking into account there was a finite population of 133 HCWs who wore white coats in the target operative units distributed as follows: obstetrics and gynecology (24), the out-patient department (14), laboratory department (16), intensive care unit (12), medical (22), pediatrics (25) and surgery (20). Therefore, the adjusted sample size, estimated according to the formula provided by Dohoo et al. [11], was 99 HCWs. We therefore targeted to 99 HWCs from distributed in the various eligible department.

### Sampling of coats

Each white coat was sampled using two sterile saline-moistened swabs with Peptone water (Oxoid). The first swab was taken from the cuff and second from the side pocket mouth. These sites were chosen because they are known to harbour microorganisms in highest concentration [12, 13]. Sampling was qualitatively done were the wet swab was rubbed against the coats and the swab was placed back into a sterile contained. Sampling was done after a written consent from the participants. The samples were labeled and transferred to the medical microbiology laboratory located with the UTH in cool boxes (+4 °C). All laboratory analyses were carried out within 1 h of sample collection.

### Culture and drug susceptibility testing

The swabs collected were directly inoculated in duplicate on blood agar, (Oxoid) Mac Conkey agar and Muller Hinton agar (Oxoid). The pairs of inoculated media were incubated aerobically at 37 °C for 24 h and then examined for bacteria growth according to standard protocol [14]. The positive culture growths were examined for colony characteristics, Gram-reaction and biochemical characteristics as described by Cheesbrough (2000) [14]. The biochemical characteristics tested were: (i) catalase, (ii) coagulase, (iii) oxidase, (iv) hemolysis, (v) sugar fermentation, (vi) indole production, (vii) citrate utilization, (viii) urease activity, (ix) triple sugar iron and (x) hydrogen sulphide production.

Purified bacterial isolates were subjected to drug susceptibility tests using the Kirby Bauer disc diffusion method [14]. Commercially available antibiotic discs that were used include: ciprofloxacin, norfloxacin, gentamycin, erythromycin, clindamycin, cephalexin, co-trimoxazole, tetracycline, cefoxitin, ampicillin, vancomycin and chloramphenicol (Hi Media Laboratories Pvt. Ltd, India). These antibiotics were chosen based on the type of microorganisms frequently isolated and their availability at University Teaching Hospital.

### Collection of epidemiological data

A pretested structured questionnaire was used to collect data needed for evaluation of the relationship between white-coat contamination and usage/handling practices by health workers. Participants interviewed were drawn from different medical specialties and units listed above and only those persons who volunteered to participate were enrolled into the study. The data collected included information on demographics, history, and duration of usage and personal hygiene practices. Each participant completed an anonymous study questionnaire soliciting information regarding his or her specialty/unit, and white-coat usage practices (e.g., length of usage, frequency of washing, number of white coats possessed, type of cleaning agents used and frequency of usage in the hospital). For the purpose of this study, the usage of a white coat was defined as the approximate length of time the health workers wore the coat while on duty.

### Data analysis

After collection, the data was coded and was checked for completeness, and the Statistical Package for Social Sciences (SPSS®) version 16.0 was used to process and analyze the data. A coat was considered contaminated if any bacterial was isolated on culture regardless of quantity. Frequency tables and other statistical presentations were done using Microsoft Excel® 2007. The Chi-square’s exact test was used to test for associated between categorical variables and the outcome of interest (coat contamination); and also between two predictor variables. The p-value of less than 0.05 was considered as statistically significant.

### Ethics approval

Ethical approval was sought from the University of Zambia, School of Medicine Research Ethics Committee and written consents were obtained from all the study participants.

## Results

A total of 107 health care workers participated in this study and had their coats sampled. The distribution of workers whose coats were sampled, according to work department is as follows: obstetrics and gynecology (18), the out-patient department (10), laboratory department (15), intensive care unit (9), medical (20), pediatrics (20) and surgery (15).

Of the 107 white coats screened, 94 (72.8 %) were contaminated with bacteria. There was no difference in the contamination levels between white coats worn for more than 60 min (47.8 %) compared to those worn for 30–60 min (46.7 %) (*p* = 0.612). Further, there was no statistical difference in contamination levels by number of coats owned. Similarly, there was no statistical difference in contaminations between coats washed once a week compared to those washed more than once (*p* = 0.7). Contamination of coats among those who used disinfectants was slightly lower (33.3 %) compared to those never did (58.1 %), although this was not statistically significant (*p* = 0.1). Coats stored at home were more likely to be contaminated (62.9 %) compared to those stored within the hospital premises (26.7 %) (*p* = 0.03). On the other hand, white coats worn just within departments were more likely to be contaminated (52.7 %) compared to those worn both inside and outside the respective department (36.4 %). Coat contamination rate by type of usage was higher for those used in clinical duties only than for those used in both clinical and non-clinical duties (Table 1).

**Table 1:** Relationship between white-coat usage/handling practices and bacterial contamination of white coats among health workers (*n* = 107) at UTH (2014)

Parameter assessed	No. of white coats examined	No. of white coats contaminated	Contamination rate (%)
Length of time of white coat is in use			
30–60 mins	15	7	46.7
>60 mins	92	44	47.8
Total	107	51	47.7
Number of white coats possessed			
1	18	8	44.4
2	49	23	46.9
>2	40	20	50.0
Total	107	51	47.7
Frequency of laundering of white coat per week			
Once	29	12	41.4
>Twice	78	39	50.0
Total	107	51	47.7
Places worn			
Within department	74	39	52.7
Outside department	33	12	36.4
Total	107	51	47.7
Professional			
Medical officer	83	39	47.0
Lab personnel	24	12	50.0
Total	107	51	47.7
Gender			
Male	85	42	49.4
Female	22	9	40.9
Total	107	51	47.7
Storage			
Within hospital	45	12	26.7
Home	62	39	62.9
Total	107	51	47.7
Disinfectant used			
Yes	45	15	33.3
No	62	36	58.1
Total	107	51	47.7

As measured by profession, coats worn by labaratory officers were observed to have slightly higher contamination rates (50 %) than medical officers (47 %). Contamination rates of coats worn by male health workers was observed to be higher (49.4 %) as compared to those worn by female health workers (40.9 %).

The results further showed that *Staphylococcus aureus* contamination had the highest isolation frequency (17.8 %) followed by *Pseudomonas spp*. (3.7 %) whereas *E. coli* and *Enterobacter spp.* had the least isolation frequencies of 0.9 % each. Gram negative bacterial (GNB) were isolated from 5.6 % of the coats.

### Drug susceptibility testing

The antibiotic sensitivity tests indicated that the bacterial isolates were resistant to some of the antibiotics assessed. Isolates of *Staphylococcus aureus* and *Klebsiella pnumoniae* exhibited the highest resistance to most of the antibiotics assessed. The most effective antibiotics were ciprofloxacin, chloramphenicol and gentamycin. Clindamycin, cotrimozaxole, ampicilin and cefoxitin exhibited some measures of resistant against GNB, *Pseudomonas spp, Escherichia coli, Klebsiella pnumoniae* and *Staphylococcus aureus,* respectively (Table 2).

**Table 2:** Antimicrobial susceptibility test of bacterial isolates from white coats

Antibiotics	*S. aureus*			*K. pneumoniae*			*Pseudomonas spp.*			*E. coli*			*Enterobacter* spp.			GNB			Total		
	*N*	*R*	%R	N	R	%R	N	R	%R	N	R	%R	N	R	%R	N	R	%R	N	R	%R
Ciprofloxacin (5 mcg)	3	1	33				4	1	25	2	0	0	4	2	50	6	1	16.7	19	5	26.3
Norfloxacin (10 mcg)	13	6	46.2																13	6	46.2
Gentamycin (10 mcg)	4	2	50	2	0	0	4	1	25	2	0	0	4	2	50	6	1	16.7	22	6	27.3
Erythromycin (15 mcg)	19	10	52.6																19	10	52.6
Clindamycin (10 mcg)	19	8	42.1																19	8	42.1
Cefalexin (30 mcg)	4	2	50	2	1	50	4	4	100	2	2	100	4	3	75	6	5	83.3	22	17	77.3
Co-trimoxazole (25 mcg)	19	13	68.4	2	2	100													21	15	71.4
Tetracyclin (30 mcg)	4	2	50	2	1	50	4	1	25	2	0	0	4	3	25	6	4	66.7	22	11	50
Chloramphenicol (30 mcg)	19	3	15.8	2	1	50	4	1	25	2	0	0	4	2	50	6	2	33.3	37	9	51.5
Cefoxitin (30 mcg)	19	13	68.4																19	13	68.4
Vancomycin (30 mcg)	19	11	57.9	2	0	0													21	11	57.9
Ampicillin (10 mcg)	4	3	75				4	3	75	2	1	50	4	1	25	6	4	66.7	20	12	60.0

*Pseudomonas spp*. and *E. coli* were both highly resistant against cephalexin (100 %). *Enterobacter spp*. and most GNB were also highly resistant against cephalexin (75 and 83.3 %), respectively. Similarly, *S. aureus* was highly resistant against ampicillin (75 %), then co-trimoxazole and cefoxitin at 68.4 % each. The least resistance was observed against chloramphenicol (15.8 %). *Klebsiella Pneumoniae* was highly resistant to co-trimoxazole (100 %), with 50 % resistance observed against cephalexin, tetracycline and chloramphenicol, respectively (Table 2).

## Discussion

The aim of this study was to establish empirical evidence related to HAIs in Zambia. This study has shown that white coats used by health care workers at the University Teaching Hospital generally harbor substantive loads of bacterial agents and could play an important role in the transmission of nosocomial infections in healthcare settings.

This study has also demonstrated that up to 72.8 % of the white coats screened were contaminated with bacteria. This result was similar to what was observed elsewhere, where white-coats contamination ranged from 23 to 95 % [15, 16]. The rate of bacterial contamination of health care workers’ white coats may be associated with patients’ continuously shedding off infectious microorganisms in the hospital environment. Other studies have suggested that a patient’s skin can be a source of contamination for the health care workers’ white coat [17, 18]. As the health care worker attends to patients, there is great possibility of cross-contamination. It has been demonstrated that microorganisms can survive between 10 and 98 days on most fabrics found in hospitals, such as those used for white coats, as well as cotton and polyester, or polyester materials [19]. In line with above research reports, this study has shown that health care workers’ white coats could contribute to the transmission of pathogenic microorganisms in a hospital environment.

The white coats of health care workers from the Paediatrics and Medical units were more contaminated than those of health care workers from the surgical wards. This study has also shown that *staphylococcus aureus,* contamination of white coats in medical, paediatrics and surgical ward was 3 % in all these units. However, Wong et al. [13] and Srinivasan et al. [20], reported that *Staphylococcus aureus* was less likely to be isolated from the white coats of health workers in a medical unit than those from a health worker in surgical or other units which was attributed to lower patient contact in the medical wards as compared to other units. However, based on the findings in this study, the low level of contamination in surgical wards could be attributed to the presence of infection prevention and control strategies at the time the study was undertaken. The medical officers’ white coats were the least contaminated compared to that of laboratory officers. Although the difference in the levels of contamination was not statistically significant, the fact that laboratory officers deal with patients’ specimens directly could explain this apparent difference. Furthermore, the laboratory is likely to harbor a high concentration of organisms within the environment since it is the place where specimen containers are opened and processing of cultures is done.

Lower rates of white-coat contaminations were observed among health workers who laundered their coats daily with disinfectants, while higher rates were observed in those who used coats for more than 60 min compared to those used between 30 and 60 min. These results were similar to what other studies had observed in the similar arrangement [15, 20]. In this study, higher contamination rates were observed in white coats used only during clinical duties when compared to those used both during clinical and non-clinical duties, suggesting a strong relationship between coat usage and patient care management. There has been controversy over whether white coats should be worn in nonclinical areas such as the canteen and libraries. This is beyond the scope of this study since transmission of infection from contaminated coats to other people was not investigated. However, it is suffice to say wearing contaminated coats could pose a risk of nosocomial infections to susceptible individuals.

*Staphylococcus aureus* and some Gram-negative bacilli were the most frequently isolated microorganisms from the white coats of health workers’ in this study. This was similar to the spectrum of bacterial agents isolated in similar investigations [16, 20]. These microorganisms are frequently found in the hospital environment and are mainly skin commensals, but they have also been implicated as causative agents of nosocomial infection [21]. In one study, it was reported that up to 65 % of nurses had methicillin-resistant *Staphylococcus aureus* (MRSA) contaminated uniforms after being in contact with patient’s wound during care activities [17]. Generally, health care workers’ hands are the other principal source of white-coat contamination with pathogens frequently found in the hospital environment. A study has shown that compliance with hand-hygiene protocols among health workers was poor in some health facilities [22].

In this study, *Staphylococcus aureus* and *Klebsiella pnumoniae* exhibited the highest rates of resistance against ampicillin and co-trimoxazole, respectively. *Escherichia coli, Pseudomonas spp., Enterobacter spp.* and some GNB exhibited resistance against cephalexin. The high levels of antibiotic resistance exhibited by bacterial isolates from the coats in this study are of concern to public health [23]. These antibiotic-resistant microorganisms are particularly important because they are capable of initiating severe nosocomiasis in a hospital environment which often require contact isolation and aggressive treatment to prevent their spread [21, 24].

## Conclusion

This study has shown that white coats worn by health workers at the University Teaching Hospital generally have high microbial contaminations, especially those worn by laboratory personnel and those worn for a longer time. This study has further established that there is a relationship between white-coat usage/handling practices and bacterial contamination, suggesting that the manner in which the white coat is used or handled by a health worker can largely determine the likelihood of its harboring and potentially transmitting pathogens. It is therefore, recommended that white coats be regularly sanitized, and health care workers also be sensitized on public health risk of HAIs associated with contaminated coats.

## References

[1] Priya H, Acharya S, Bhat M, Ballal M (2009). Microbial contamination of the white coats of dental staff in the clinical setting. J Dent Res Dent Clin Dent Prospects.

[2] Pittet D, Donaldson L (2005). Clean care is safer care: A worldwide priority. Lancet.

[3] Levy MM, Fink MP, Marshall JC, Abraham E, Angus D, Cook D (2003). 2001 sccm/esicm/accp/ats/sis international sepsis definitions conference. Intensive Care Med.

[4] Boucher HW, Talbot GH, Bradley JS, Edwards JE, Gilbert D, Rice LB (2009). Bad bugs, no drugs: no ESKAPE! an update from the infectious diseases society of America. Clin Infect Dis.

[5] Weist K, Pollege K, Schulz I, Rüden H, Gastmeier P (2002). How many nosocomial infections are associated with cross-transmission? A prospective cohort study in a surgical intensive care unit. Infect Control Hosp Epidemiol.

[6] Saloojee H, Steenhoff A (2001). The health professional’s role in preventing nosocomial infections. Postgrad Med J.

[7] Pittet D, Allegranzi B, Boyce J, World Health Organization World Alliance for Patient Safety First Global Patient Safety Challenge Core Group of Experts (2009). The World Health Organization guidelines on hand hygiene in health care and their consensus recommendations. World Health.

[8] Wiener-Well Y, Galuty M, Rudensky B, Schlesinger Y, Attias D, Yinnon AM (2011). Nursing and physician attire as possible source of nosocomial infections. Am J Infect Control.

[9] National Nosocomial Infections Surveillance System (2004). National Nosocomial Infections Surveillance (NNIS) System Report, data summary from January 1992 through June 2004, issued October 2004. Am J Infect Control.

[10] Pinon A, Gachet J, Alexandre V, Decherf S, Vialette Ml. Microbiological Contamination of Bed Linen and Staff Uniforms in a Hospital. *Advances in Microbiology*. 2013: 2013.

[11] Dohoo IR, Martin W, Stryhn HE. Veterinary epidemiologic research. 2003.

[12] Loh W, Ng VV, Holton J (2000). Bacterial flora on the white coats of medical students. J Hosp Infect.

[13] Fang G, Keys TF, Gentry LO, Harris AA, Rivera N, Getz K (1993). Prosthetic valve endocarditis resulting from nosocomial bacteremia: a prospective, multicenter study. Ann Intern Med.

[14] Cheesbrough M. *District laboratory practice in tropical countries*. Cambridge university press. 2006.

[15] Treakle AM, Thom KA, Furuno JP, Strauss SM, Harris AD, Perencevich EN (2009). Bacterial contamination of health care workers’ white coats. Am J Infect Control.

[16] Pilonetto M, Rosa EAR, Brofman PRS, Baggio D, Calvário F, Schelp C (2004). Hospital gowns as a vehicle for bacterial dissemination in an intensive care unit. Braz J Infect Dis.

[17] Boyce JM, White RL, Spruill EY (1983). Impact of methicillin-resistant Staphylococcus aureus on the incidence of nosocomial staphylococcal infections. J Infect Dis.

[18] Muto CA, Jernigan JA, Ostrowsky BE, Richet HM, Jarvis WR, Boyce JM (2003). SHEA guideline for preventing nosocomial transmission of multidrug-resistant strains of Staphylococcus aureus and enterococcus. Infect Control Hosp Epidemiol.

[19] Chacko L, Jose S, Isac A, Bhat KG (2003). Survival of nosocomial bacteria on hospital fabrics. Indian J Med Microbiol.

[20] Srinivasan A, Wolfenden LL, Song X, Mackie K, Hartsell TL, Jones HD (2003). An outbreak of Pseudomonas aeruginosa infections associated with flexible bronchoscopes. N Engl J Med.

[21] An Expert A, Healthcare P, Lewis S (2010). The potential for nosocomial infection transmission by white coats used by physicians in Nigeria: implications for improved patient-safety initiatives. World Health Popul.

[22] Harris AD, Samore MH, Nafziger R, DiRosario K, Roghmann MC, Carmeli Y (2000). A survey on handwashing practices and opinions of healthcare workers. J Hosp Infect.

[23] Jones RN (2003). Global epidemiology of antimicrobial resistance among community-acquired and nosocomial pathogens: a five-year summary from the SENTRY Antimicrobial Surveillance Program (1997–2001). Semin Respir Crit Care Med.

[24] Lee N, Lee H, Ko N, Chang C, HsinΓÇÉI Shih MD, Wu C (2007). Clinical and economic impact of multidrug resistance in nosocomial Acinetobacter baumannii bacteremia. Infect Control Hosp Epidemiol.

